# “Counteract the gaslighting” – a thematic analysis of open-ended responses about what women survivors of intimate partner sexual violence need from service providers

**DOI:** 10.1186/s12905-024-02943-1

**Published:** 2024-02-09

**Authors:** Síofra Peeren, Elizabeth McLindon, Laura Tarzia

**Affiliations:** 1 Section of Women’s Mental Health, Institute of Psychiatry, Psychology and Neuroscience, King’s College London, London, UK; 2 Service User Research Enterprise, Institute of Psychology, Psychiatry and Neuroscience, King’s College London, London, UK; 3 Department of General Practice, The University of Melbourne, Melbourne, VIC Australia; 4 Centre for Family Violence Prevention, The Royal Women’s Hospital, Parkville, VIC Australia

**Keywords:** Intimate partner violence, Sexual violence, Intimate partner sexual violence, Thematic analysis, Qualitative, Disclosure, Help-seeking

## Abstract

**Background:**

Intimate partner sexual violence (IPSV) is a prevalent but misunderstood form of gender-based violence with significant impacts women’s health and well-being. Research suggests that IPSV has a specific context and unique impacts, but little is known about how to tailor service responses. To address this gap, we explored help-seeking experiences and needs among IPSV survivors after disclosure.

**Methods:**

This study draws on qualitative data from a subsample of women who participated in a cross-sectional survey about the service needs of intimate partner violence survivors. Women who reported IPSV and provided information about IPSV-specific help-seeking needs after disclosure were included in the analysis. Open-ended text responses of 37 IPSV survivors were analysed using thematic analysis.

**Results:**

IPSV was invisible and silenced in service responses. Three themes suggest potential ways forward. In the first theme, ‘Don’t dismiss it’, women needed providers to take their disclosures seriously and listen to the significant impacts of IPSV on their well-being and safety. In the second theme, ’See the bigger picture’, women needed service providers to understand that IPSV fits into broader patterns of abuse, and that psychological abuse and coercive control impacts women’s ability to consent. In the third theme, ‘counteract the gaslighting’, women needed providers to educate them about the continuum of IPSV and help them label IPSV as a form of violence.

**Conclusions:**

Our exploratory findings extend the limited evidence base on IPSV and highlight a need for further in-depth research to explore a tailored approach to supporting IPSV survivors. To avoid contributing to the silencing of IPSV survivors, service responses should recognise the harmful and sexualised nature of IPSV, challenge cultural stereotypes that minimise IPSV, and understand that co-occurring psychological abuse may exacerbate shame and prevent women from articulating the source of their distress.

## Background

Intimate partner sexual violence (IPSV) is a common yet hidden form of violence against women [1, 2]. Sitting at the intersection of intimate partner violence (IPV) and sexual violence (SV), IPSV is defined as any sexual act performed against a person’s will by a current or previous partner [1, 3]. IPSV profoundly impacts women’s health and well-being, and may lead to mental distress, suicide attempts, miscarriages, and sexually transmitted infections [4]. Although extant research focuses on partner rape and sexual assault, IPSV includes a broad spectrum of behaviours including verbal coercion and threats to obtain sex, pressure to use pornography, and sexually degrading comments [1, 5–7]. Although robust prevalence data is lacking, studies estimate that between 10% and 22% of women in Australia and the United States have been subjected to IPSV [8–10]. However, the true prevalence is likely to be much higher as all forms of violence against women and girls are grossly under-reported and IPSV is a particularly stigmatised and silenced form of violence [2, 11].

Despite IPSV’s high prevalence and significant health impacts, research on service responses to IPSV is scarce [6]. The World Health Organisation recommends using the LIVES model to respond to IPV or non-partner SV (listening, inquiring about needs, validating experiences, enhancing safety and offering ongoing support) [12]. For IPV, the CARE model (choice and control, action and advocacy, recognition and understanding, and emotional connection) has been developed to be used alongside LIVES to guide health practitioners to deliver woman-centred care [13]. Additionally, a recent review highlighted that women value education from healthcare providers to help them identify IPV and seek specialist support, irrespective of disclosure [14]. These studies highlight the critical importance of service providers responding in ways that show empathy for and directly address the dynamics of IPV. However, despite evidence of increased help-seeking among IPSV survivors [3], very little is known about how to respond to IPSV, with guidance for service providers outside of healthcare particularly lacking [4].

Although many of the basic principles of a trauma-informed response are similar across different forms of violence against women, it is increasingly recognised that responses need to be tailored to the individual needs of survivors. Qualitative research strongly suggests that the dynamics and context of IPSV are different to those for other types of IPV or non-partner sexual assault [6, 15]. The acute betrayal of being abused by someone who is meant to love them and the sexualised nature of the violence means IPSV is experienced by survivors as a particularly degrading and dehumanising form of violence, setting it apart from other forms of IPV and non-partner SV [2]. Sexual violence literature more broadly emphasises that providers should avoid subscribing to ’rape myths’ [16]. However, unique elements of IPSV highlighted by qualitative research signal the importance of more targeted support from service providers. For example, in IPSV, cultural myths intersect with gendered ideas around women’s duties in relationships and co-occurring psychological abuse to facilitate and normalise violence and compound shame [6, 15]. Furthermore, IPSV survivors may experience subtle, psychologically coercive behaviours that are much more difficult to label as violence but no less harmful to their well-being and safety [2]. However, no study to date has examined help-seeking needs from the perspective of IPSV survivors. Addressing this gap, we explored what women need from service providers after disclosing IPSV from survivors’ perspectives. The guiding question for this research was, ‘what do women need from professionals when seeking support for IPSV?’.

## Methods

### Materials and methods

This study reports on a subsample of women who participated in a cross-sectional survey (VOICES) about patterns of intimate partner violence and the service needs of survivors [17]. Women were included in our analysis if they reported IPSV and provided information about what they needed from service providers after disclosure of IPSV. We initially intended to include the responses of all women who reported IPSV (n = 409). However, when we examined the data on help-seeking, we found that responses did not always specify the type of violence that women were referring to. As many participants had had multiple experiences, we therefore only included responses that referred specifically to IPSV to ensure the findings reflected IPSV-specific needs. Therefore, our final sample is women who reported IPSV and made specific comments about IPSV-related help-seeking (n = 37).

Questions about IPSV experiences included: [1] Has a current or previous partner ever physically forced you to have sex when you did not want to?; [2] Have you ever had sex with a current or previous partner when you did not want to because you were afraid of what he might do?; [3] Has a current or previous partner ever forced you to watch pornography when you did not want to?; [4] Has a current or previous partner ever forced you to do something else sexual that you did not want to? The team developed the IPSV questions specifically for the VOICES survey.

Service providers were defined as those offering to assist people in areas of general psychosocial need (housing, financial); health/counselling (general practitioner, nurse, psychologist/counsellor/family therapist, social worker, alcohol or drug worker, general telephone helpline, religious leader); specialist IPV support/advocacy (IPV, SV service); or law enforcement/legal response (police, legal service). Piloting the survey led to modifications of both the wording and the pathways through the survey. Participants answered the following open-ended survey questions about service needs: [1] What do you wish providers had done differently after speaking to them about your partner’s behaviours? and [2] What do you wish providers had done differently after speaking to them about unwanted sexual encounters?

### Recruitment and data collection

The online survey was anonymous and voluntary and conducted between January 27th 2021 and January 31st 2022. Participants were recruited from across Australia, initially via a link promoted through traditional and social media channels, followed by the employment of a third-party research company i-Link to address difficulties in reaching the target sample size. I-Link is a social and market research company that administers online surveys to panel members across Australia [18].

### Ethics

The survey was approved by The University of Melbourne’s Human Research Ethics Committee (ID number: 2,057,459). Participants were asked to read an Information Sheet prior to beginning the survey and consent to participate was indicated through returned surveys. We followed guidelines on ethical research with survivors of interpersonal violence, including that research must be trauma-informed, protect safety and maximise choice and control over research involvement [19, 20]. Survey questions were optional to recognise their sensitive nature and to maximise choice and control. Each section began with a description of the topics the questions would cover so that participants knew what to expect. To protect safety, contact details for support services were provided throughout the survey and each page contained a quick exit link. The survey took approximately 25 to 30 min to complete. Participants recruited via social media received a small gift voucher as a token of appreciation and the i-Link participants were remunerated via their panel.

### Approach to analysis

We used reflexive thematic analysis to analyse the data [21, 22]. Reflexive thematic analysis is particularly well suited to applied research topics where there is minimal prior knowledge and when exploring how personal experiences sit within broader socio-cultural contexts [21, 22]. The following decisions guided the analysis [21, 22]: prioritising salience over the prevalence of themes; focusing on one aspect of the dataset (i.e. service responses to IPSV); taking a data-driven, inductive approach (recognising that this is often a continuum rather than a dichotomy); and identifying themes based on the explicit or surface (semantic) meaning of the data rather than looking for underlying or unsaid assumptions (latent meaning). We aimed to produce findings that may be useful for practice. Therefore, in our analysis, we assumed that the data and findings reflect some version of reality or ‘truth’ whilst recognising that this reality is socially-mediated and the findings are shaped by both the researchers and the research context [23–25]. NVivo12 was used for data management [26].

Codes were generated by the first author (SP) and refined through regular discussions with the last author (LT). All authors (SP, EM and LT) were involved in developing the final themes and discussed their interpretation at length to ensure rigour and accurate representation of the data. The steps taken in the analysis followed guidance by Braun and Clarke [21]:

1. The first analysis phase involved familiarisation with the data through reading and re-reading to become immersed in its content.
2. The next phase involved generating codes that identified important features of the data. These codes were later collated into initial themes.
3. Codes were then examined to identify key patterns across the dataset and to generate initial themes. To do this, the codes within each theme were reviewed to judge the salience of each potential theme. We distinguished between codes and themes by drawing on the following definition: “codes can be thought of as entities that capture (at least) one observation, display (usually just) one facet; themes, in contrast, are like multi-faceted crystals” [21, p. 340]. All themes represented a pattern in the data and were present across multiple accounts, but as we chose themes based on what we judged to be their importance [21] this pattern could be small.
4. Next, potential themes were checked against the data to ensure they reflected their content and answered the research question. At this stage, themes were further refined if necessary, which involved working out each theme’s central idea, scope and focus.
5. Finally, themes were refined and finalised to ensure they reflected the content of the data and formed a coherent narrative.

### Researcher positionality and reflexivity

Reflexive thematic analysis is grounded in an interpretive methodology that acknowledges and celebrates researcher positionality [22]. It is therefore important to state our assumptions and background here. All authors consider IPSV to be rooted in gender inequity and work from the premise that service/health systems responses are a critical aspect of the community response to gender-based violence. The first author has lived experience of IPSV and help-seeking for IPSV as well as extensive experience of conducting qualitative research with survivors of interpersonal violence. Her experiential and research knowledge informed the analysis through engaging in an ongoing process of reflexivity. The second and third authors are researchers with backgrounds in social work and sociology, respectively; both have extensive expertise in the area of sexual violence. The second author also works as a sexual assault counsellor/advocate.

## Results

Responses varied in length and level of detail. The longest response was 130 words, whilst the shortest was 7 words. The majority of participants wrote answers of at least 20 words (around 2 sentences). Table 1 summarises participant characteristics. All participants (n = 37) identified as women. Most participants were aged 45 years or less (n = 36), had been born in Australia (n = 28), spoke English as a first language (n = 35) and lived in a metropolitan area (n = 31). Fifteen had a current partner, and most partners (n = 13) were male. The majority of participants had children (n = 32) and had completed Year 12 (n = 31). Just over half had a university degree (n = 22) and were healthcare card holders (n = 22). In addition, approximately two thirds of participants were employed (n = 25) and a similar number found their income difficult to manage some or all of the time (n = 24).

**Table 1:** Demographic characteristics of the 37 survivors of intimate partner sexual violence who provided open-ended VOICES survey data about the needs they have of service providers

Characteristic	No. (%) of participants
**Age (years)** (*n* = 37)	
Up to 25 years	19 (51.3)
25–45 years	17 (45.9)
> 45 years	1 (2.7)
**Aboriginal/Torres Strait Islander** (*n* = 37)	0
**Born outside Australia** (*n* = 37)	9 (24.3)
**First language not English** (*n* = 37)	2 (5.4)
**Current Australian state of residence** (*n* = 37)	
Australian Capital Territory	2 (5.4)
New South Wales	8 (21.6)
Victoria	8 (21.6)
Northern Territory	1 (2.7)
Queensland	10 (27.0)
Western Australia	1 (2.7)
Tasmania	1 (2.7)
South Australia	6 (16.2)
**Lives in a metropolitan area** (*n* = 37)	31 (83.8)
**Lives in non-metropolitan area** (*n* = 37)	6 (16.2)
**Lives with a partner** (male/female/spouse) (*n* = 37)	6 (16.2)
**Has current partner** (*n* = 37)	15 (40.5)
**Sex of current partner** (*n* = 15)	
Male	13 (86.7)
Female	2 (13.3)
**Children** (*n* = 37)	
1 + children living at home	32 (86.5)
**Prior schooling** (*n* = 36)	
Attended school but finished prior to year 12	5 (13.9)
Completed year 12	31 (86.1)
**Educational qualification** (*n* = 36)	
Trade/Apprentice/Diploma	14 (38.9)
University degree/higher degree	22 (61.1)
**Income & employment** (*n* = 36)	
Employed	25 (69.4)
Not in employment	11 (30.6)
Finds income difficult to manage some/all time	24 (66.7)
Healthcare card holder	22 (61.1)

Table 2 summarises the themes and the central idea of each theme. Each theme captures an element of what women IPSV survivors said they needed from service providers after disclosing IPSV: (1) Don’t dismiss it; (2) See the bigger picture; (3) Counteract the gaslighting.

**Table 2:** Overview of theme names and the central idea of each theme

Theme	The central idea of the theme
Don’t dismiss it	Providers minimised disclosures when they assumed SV should involve physical force, that consent does not apply in a relationship, or that sex is part of a woman’s duty. Normalising IPSV led to continued and escalated violence for some women since their partner’s behaviours were not considered dangerous. Women needed providers to take their disclosures seriously and genuinely listen to how their partners’ behaviours impacted them
See the bigger picture	Women understood psychological abuse to be an important part of IPSV. IPSV could be part of a pattern of psychological abuse and psychological abuse could facilitate IPSV. Links between psychological abuse and IPSV led to women feeling violated without understanding or being able to articulate why. Women wanted practitioners to recognise IPSV within broader patterns of control and help women identify the source of their distress
Counteract the gaslighting	To address the silencing of IPSV, women wanted providers to believe them and counteract the shame and disorientation they felt. Women also wanted service providers to help them identify IPSV and seek support for IPSV. Service providers needed to educate women about the context of IPSV, help women label IPSV as a form of violence, and challenge cultural stereotypes that minimised and perpetuated IPSV

### Don’t dismiss it

This theme focuses on women’s understanding of why service providers minimised their disclosures and the impact that this had on them. Women reported that when they disclosed their partner’s sexually coercive behaviours, service providers generally believed that the behaviour had happened but denied that it was violent. Women identified several cultural assumptions that led to IPSV being minimised and women being blamed. For example, many women reported experiencing psychological manipulation that put pressure on them to ‘agree’ to sex, such as partners repeatedly asking for sex or using threats. Women noted that service providers often believed that their partners were using these behaviours but the lack of physical violence led them to assume that these behaviours were neither serious nor violent. Here, service providers made assumptions about the severity of the behaviours instead of looking at the pattern of the behaviours or listening to the impact that these behaviours had on women.

My ex-partner would threaten to kill himself if I wouldn’t have sex with him. None of the professionals I’ve spoken to seem to think that’s a big issue (Participant 32).

Women reported how minimising responses from service providers reinforced cultural stereotypes that it was a woman’s duty to be sexually available to her partner and that women ‘should’ enjoy all sex with their partner. In so doing, service providers pathologised women’s distress at their partner’s sexually violent behaviours instead of questioning whether their partner’s sexually coercive behaviour was appropriate.

The counsellor I saw told me that it was my duty to satisfy and submit to my husband and were focused on teaching me how to be more responsive and give him sex happily when he wanted it (Participant 694).

[Service providers need to] realise that my husband asking for sex repeatedly when I didn’t want it might be a cause for concern rather than just try to find ways to increase my sex drive (Participant 411).

Even IPSV that involved physical violence was minimised, however. In these cases, women reported that service providers dismissed their disclosures of sexual violence either because they presumed that consent was given by virtue of being in a relationship or that consent did not apply with an intimate partner. This response told women that what they experienced was not, in fact, sexual violence, and invalidated their distress.

[Service providers should not] assume that because it’s your partner that you must have consented (Participant 497).

Professionals need to stop shaming victims, including the idea that it was okay because it was my partner (Participant 248).

Women wanted service providers to know that intimate partner sexual violence was a dangerous and serious issue. Women reported that, when those who were meant to support and help protect women did not listen, it forced them to endure continued, potentially escalated violence, leaving some women with nowhere to turn.

The police didn’t believe me, broke me. He forced me to have sex with him, forced me to sleep on the laundry floor. I went without showers he abused me for five months. Believe the victim (Participant 9).

In this theme, women described how providers did not listen to what women told them about the impact of their partner’s behaviours on their sense of safety and well-being. Instead, women noted that providers made assumptions about the severity of the behaviour based on stereotypes that excuse and minimise sexual violence from partners. Women tied these minimising responses from service providers to stereotypes about what sexual violence looks like (that it should involve physical force); who perpetrates sexual violence (strangers, not partners); and gendered ideas about women’s duties in relationships (women should be sexually available and should sexually submit to, and enjoy, all sexual acts with their partners). These stereotypes told women that their partner’s behaviours were not violent when they were, and that women’s feelings of violation and distress indicated a problem with them, not their partner.

### See the bigger picture

This theme focused on the relationship between psychological abuse and IPSV and how this impacted women’s ability to understand their partner’s behaviours as sexual violence. This theme provides context about *why* women found their partner’s behaviours so distressing, capturing how women reported feeling when presenting to services and underlining the importance of providers seeing ‘the bigger picture’. Women reported that experiencing sexual violence within a pattern of abuse was critical to their experiences of IPSV, and this also made their partner’s behaviours and their impact so challenging to understand and articulate.

Some women understood IPSV as part of a general pattern of psychologically abusive behaviours used to degrade them and erode their self-worth. These women emphasised that IPSV was part of a bigger picture and could not be fully understood by focusing on isolated incidents alone.

[IPSV] was part of the objectification and reasons for the emotional blackmail. They [service providers] need to understand that it is part of a bigger picture of controlling behaviours used to demean their partners (Participant 324).

Women also talked about how the environment of fear and control created by psychological abuse facilitated sexual violence by interfering with their ability to give enthusiastic consent. Women did not detail how psychological abuse affected consent. However, they made it clear that they wanted service providers to understand that psychological abuse impacted consent in subtle and nuanced ways that may not seem obvious.

[Service providers need to] have a better understanding of the fact that emotional abuse and controlling nature of the relationship can lead to forced sexual encounters, even though it’s not what would be deemed “rape” (Participant 250).

[Service providers need to understand] all the nuanced ways consent violations and coercive control can work in a relationship (Participant 404).

These links between psychological abuse and IPSV may explain why the behaviours women outlined in the previous theme, such as partners asking for sex repeatedly, were so distressing. Women reported that the environment of control and fear created by psychological abuse left them with no choice but to submit to their partner’s demands. Yet, this relationship between IPSV and psychological abuse, coupled with stereotypes about ‘real’ rape, disrupted women’s ability to understand and articulate exactly why they felt violated and degraded by their partner’s behaviours.

[Service providers need to] understand that a woman may have difficulty labelling an unwanted sexual encounter as rape/assault or even unwanted. A woman may indeed yearn for intimacy with her partner but end up feeling degraded and violated without truly understanding why. Particularly if her confidence has been eroded by constant critique (Participant 56).

I didn’t often seek out help because what I experienced seemed ‘not that bad’ compared to the stereotype. (It was very bad) (Participant 404).

In this theme, women wanted service providers to look at the ‘bigger picture’ and described that IPSV’s complex relationship with psychological abuse impacted women’s ability to articulate and understand their experiences. Psychological abuse could facilitate IPSV, and IPSV could also be part of an overall pattern of psychological abuse. However, regardless of the nature of this relationship for individual women, this pattern of abuse disoriented women and made them feel ashamed and violated. Women needed providers to understand and acknowledge the complex and nuanced relationships between IPSV and psychological abuse in their responses.

### Counteract the gaslighting

This theme was about how women reported needing service providers to not only believe their disclosures of IPSV but to actively shift the blame away from women and onto partners. Shifting shame and blame counteracted the silencing and erosion of self-worth that resulted from the disorienting psychological abuse women endured alongside their partner’s sexually violent behaviours and the cultural silencing of IPSV. Service providers could ‘counteract the gaslighting’ by educating women about what IPSV looked like and challenging cultural stereotypes that excused IPSV. Women reported wanting specific education about how sexual violence manifested in relationships, including how partners exploited gendered stereotypes and the relationship context to pressure women to agree to sexual acts. Many women pointed out that IPSV was shrouded in silence, stigma and shame and that, consequently, women and girls needed access to education about IPSV from service providers, irrespective of disclosure.

Women reported experiencing a wide range of sexually coercive behaviours, many of which did not align with societal stereotypes about what ‘real’ sexual violence is. Women wanted providers to send a strong message that these sometimes subtle behaviours were unacceptable and were in fact violent.

[Service providers need to] talk about what is not ok. It is not ok to badger your partner into having sex. It is not ok to have sex with them while they are asleep. It is not ok to isolate them if they say no to sex (Participant 94).

Women reported wanting providers to educate them about consent in the context of IPSV. Women needed service providers to actively challenge cultural myths that equated ‘love’ with ‘consent’. These cultural myths silenced and confused women and prevented them from having the language to label the sexually coercive behaviours as violent. Dismantling these cultural myths validated women and helped women to identify their experiences as violence and the source of their distress.

Educate me about what unwanted sexual encounters are. It was very confusing when unwanted sexual encounters occurred because I thought that it shouldn’t occur and because I understood myself to be in a supposedly consenting or ‘loving’ relationship with an intimate partner at the time (Participant 256).

Specifically, women wanted service providers to clearly communicate that psychological coercion could prevent genuine, enthusiastic consent just as much as physical violence. Doing so challenged the stereotypes that sexual violence must include physical force that intersected with the often more manipulative and covert ways partners sexually coerced women. This addressed the confusion and disorientation women felt.

Making it clear that coercion into sexual acts doesn’t have to be physical. It can be done through emotional manipulation (Participant 556).

Women also highlighted a role for universal education on respectful relationships and enthusiastic consent, particularly education that emphasised partners’ equal responsibility to ensure that the other partner is consenting. This was important because it shifted blame away from women for feeling distressed and confused by their partner’s sexually violent behaviours and onto the perpetrator for behaving in ways that prevented enthusiastic consent or ignored their responsibility to ensure consent.

[Provide] education on what a respectful relationship looks like and what enthusiastic consent is (Participant 56).

Women also reported needing service providers to challenge stereotypes about sex being part of a woman’s duty to her partner that compounded shame and excused sexual violence in a relationship. Providers who did this emphasised a woman’s choice to say no to sex no matter the circumstances whilst recognising that gendered expectations of women within relationships may make saying no more difficult.

Clearly define what is acceptable and unacceptable behaviour of a husband, and how wives are not obliged to live up to their expectations (Participant 506).

In this theme, women wanted providers to actively challenge the stereotypes attached to IPSV and counteract the impact of the psychological abuse women experienced alongside or as part of IPSV. It was important to women that providers went beyond believing women and understanding IPSV to actively shifting the blame from women onto the partners who perpetrated IPSV. To shift blame, responses from service providers needed to address the unique ways in which consent was disrupted by psychological abuse and IPSV facilitated by cultural stereotypes that blamed women. Women wanted universal education to address the specific context of IPSV irrespective of disclosure or risk factors.

## Discussion

Drawing on a thematic analysis of open-ended text responses, this research explored what women needed from service providers when seeking support for IPSV. Our findings suggest that IPSV was hidden and dismissed on multiple levels. Above all, women in our study wanted providers to treat IPSV seriously by genuinely listening to what women were telling them about their partner’s sexually violent behaviours and the significant impacts of IPSV on their sense of well-being, self-worth, and safety. Survivors reported that gendered, cultural stereotypes about women’s roles and duties in relationships created barriers to disclosure and identification and led to blaming and shaming responses from service providers that were specific to IPSV. Furthermore, co-occurring psychological abuse disoriented women, compounding shame and preventing them from understanding and articulating the reasons for their distress. Women reported needing providers to actively counteract the cultural silencing of IPSV and the impacts of the psychological abuse they endured by naming IPSV as violence and educating women about what IPSV looked and felt like. Table 3 outlines the themes generated and recommendations for practice associated with each theme.

**Table 3:** Recommendations resulting from each finding

Theme	Recommendations
Don’t dismiss it	Listen and respond to the impact of partners’ behaviours on women and avoid making assumptions about the severity of the behaviour(s). Be aware of and challenge cultural stereotypes that minimise IPSV, such as (1) women should be sexually available to their partner, (2) women should enjoy all sex with their partner, and (3) women cannot be raped or sexually assaulted by a partner
See the bigger picture	Focus on the pattern of behaviours, not on individual incidents. Understand that co-occurring psychological abuse may exacerbate shame and prevent women from articulating the source of their distress. Acknowledge that relationships between IPSV and psychological abuse are complex; psychological abuse can facilitate IPSV and IPSV can also be part of a broader pattern of fear and coercive control
Counteract the gaslighting	Actively challenge cultural stereotypes that minimise and excuse IPSV. Name all forms of IPSV as violence and abuse irrespective of whether it co-occurs with physical violence; educate women about the continuum of IPSV behaviours; acknowledge intersections between psychological abuse and IPSV and how these may disrupt consent and compound shame. Universal education about healthy relationships and enthusiastic consent may empower women with information and language they need to identify IPSV and seek support

Survivors reported that IPSV was so normalised in society that it was invisible in services. A new finding of this study, and a strong theme across the dataset, was that women described disclosures being dismissed because services considered IPSV to be neither sexual violence (SV) nor intimate partner violence (IPV), leaving women feeling isolated and imprisoned by the abuse. Women struggled to articulate why their partner’s behaviours were so distressing because they did not always have the language to identify their partner’s behaviours as sexual violence. When women disclosed, service providers mirrored perpetrators in telling women that their distress indicated something wrong with *them* rather than their partner’s behaviours. Cultural perceptions of ‘real rape’ intersected with gendered ideas about women’s roles in relationships and co-occurring psychological abuse to create significant and unique barriers to seeking and receiving appropriate support. Indeed, qualitative research has found that IPSV survivors had a strong, internalised sense of duty that came from wider society [6]. Our findings describe ways in which service providers’ responses either reinforced or challenged this sense of duty. Like IPV and SV, women understood dismissive responses from service providers to be rooted in cultural stereotypes that excuse violence and blame survivors [13, 14, 16, 27]. However, importantly, the cultural stereotypes described by women in this study were specific to IPSV and indicated that IPSV was a particularly silenced, stigmatised and misunderstood type of interpersonal violence that required a targeted response from professionals.

Women suggested several ways to overcome the silencing surrounding IPSV, all of which require service providers to thoroughly understand its unique context, impacts and dynamics. Women needed providers to understand complex and bi-directional relationships between psychological abuse and IPSV; listen to and respond to the impact of partners’ behaviours on women instead of making assumptions about severity; educate women about identifying IPSV, and actively challenge cultural stereotypes that minimise and normalise IPSV. This indicates that providers should have access to training to address personal barriers to identification and response [28]. Our findings indicate a need for specific training on IPSV for providers that teach them about the continuum of IPSV behaviours, how psychological abuse may intersect with sexual violence to disrupt consent, the multiple barriers to disclosure women face, and the cultural stereotypes weaponised by perpetrators to silence survivors. Although knowledge about IPSV is important, our findings suggest that providers should be encouraged and expected to consider IPSV as a social justice issue and human rights violation that requires a community response of which they are a critical part [29].

Another prevalent theme was that women needed providers to actively counteract the confusion and disorientation they felt due to IPSV. Other research has found that women need providers to ‘do more than just listen’, for instance, through advocating and taking action [13]. Our findings also support the universal education approach highlighted Korab-Chandler and colleagues [14]. In this study, survivors of IPV wanted education on healthy relationships and warning signs of abuse, irrespective of disclosure, to empower all women with the information and tools to identify IPV and take steps to seek support [14]. In our study, women similarly wanted service providers to empower them with information and education on respectful relationships and warning signs of IPSV. However, our findings emphasise that for such education to help women identify and seek support for IPSV, it must directly target its uniquely shaming, silencing and disorientating context. Therefore, our findings suggest that education should address enthusiastic consent, intersections between psychological abuse and IPSV, and the multiple and complex barriers women face in labelling IPSV as violence. Importantly, education needs to challenge cultural stereotypes specific to IPSV, including ideas that sexual violence must include physical violence, that consent is assumed or does not apply in a relationship, or that women have a duty to be sexually available to their partner. Furthermore, recognising that these stereotypes manifest in complex, nuanced ways that can be very confusing for women is critical.

Our findings contribute to understandings about intersections between IPSV and psychological abuse – a relationship that until recently was not well understood [10, 15]. Women in our study reported that co-occurring psychological abuse prevented them from identifying and voicing their experiences as sexual violence. Although this theme was smaller than the others, it is an important one as it captured intersections between psychological abuse and intimate partner sexual violence. These have rarely been explored in the literature but appear to be central to understanding the multiple layers of silencing that IPSV survivors in our study experienced. A recent qualitative study explored complex ways that psychological abuse and IPSV were related and worked to erode women’s self-worth, highlighting that relationships are more complex than previously thought [15]. The voices of the women included in this study offered specific ways that service providers could begin to address the intersection between psychological abuse and IPSV, although further research is needed. For instance, women needed service providers to counteract the disorientating environment created by co-occurring psychological abuse and IPSV by empowering women with language and information they need to name and identify IPSV.

### Strengths and limitations

Subjectivity is celebrated as a strength within qualitative paradigms, provided researchers take steps to reflect on the socially situated and experiential knowledge that they may bring to the analysis [22]. As the first author was herself a survivor of IPSV, the analysis was survivor-led and conducted from a perspective borne of lived experience. Conducting research from an explicit survivor standpoint may increase the likelihood that findings will resonate with survivors [30], a goal which lies at the heart of trauma-informed approaches [31]. A related strength was that trauma-informed research design principles were incorporated into the survey design [20]. Additionally, IPSV survivors were identified using questions about behaviours often excluded from IPSV definitions, such as being forced to watch pornography [32]. A large group of diverse participants participated in the parent survey, although the survey was only available in English and to those with internet access [17]. The anonymity of surveys may also enable participants to share their experiences of highly stigmatised and silenced topics more freely [25].

The online nature of the survey meant that we could not verify if participants had done the survey more than once. However, this was mitigated by checks for duplication of data. The implications that can be drawn from our study are limited by its exploratory nature. Although surveys are considered a valid method of identifying and analysing patterns of meaning [25], using open-ended text responses may have limited the richness of data. Furthermore, the lack of IPSV-specific questions about help-seeking reduced the pool of eligible responses. As most women had had multiple experiences, we could only include responses that specifically mentioned IPSV. As a result, we may have missed responses that referred to IPSV-specific help-seeking needs but did not explicitly mention IPSV. Although our data may lack richness, our findings set a foundation for further qualitative research in this under-researched area.

### Further research

This research provides an initial understanding of what IPSV survivors may need from service providers after disclosing IPSV. However, further in-depth qualitative research is needed in order to develop these findings. Considering the stigmatised, hidden and disorientating nature of IPSV, further research could usefully examine women’s pre-disclosure experiences to understand the facilitators and barriers to identifying and seeking support for IPSV. The women in this study also highlighted several barriers to responding to IPSV at the service provider and system levels, such as cultural assumptions that consent does not apply in a relationship. Further research is needed to understand what support service providers may need to provide the tailored, trauma-informed response required to fully address IPSV. Finally, further research is needed to explore how survivors’ sociodemographics and intersecting identities may shape their experiences of services. For survivors in our study, harmful responses to disclosures were often gendered and rooted in sexist ideas about women’s duties, but due to its qualitative and exploratory nature we could not explore differences between particular groups of participants. It is critical for future research to explore how sexism may intersect with other systems of oppression, such as racism, classism and ableism, to create additional layers of silencing for some survivors.

We included two open-ended questions in this analysis; the first asked women about what they needed from services after disclosing intimate partner violence, and the second asked women what they needed from service providers after disclosing sexual violence. Neither question asked explicitly about IPSV, yet both questions were relevant to IPSV – an experience that is both IPV and SV. Most participants only described IPSV in response to the SV-specific help-seeking question, referring almost exclusively to non-sexual forms of IPV (e.g., physical and psychological violence) in response to the IPV-specific help-seeking question. This pattern may reflect the silenced and taboo nature of IPSV and that women may not disclose IPSV unless specifically asked about sexual violence. This suggests an avenue for further research to explore what might help survivors disclose IPSV.

## Conclusion

The findings of this study extend the extremely limited evidence base about what women need from service providers when seeking support for IPSV. Although further in-depth qualitative research is needed, these findings provide a first step towards understanding how providers can address the unique context and impacts of IPSV in their practice and highlight the devastating impacts on women when disclosures remain unheard. Our findings highlight the need for a specific and tailored approach that recognises the cultural silencing of IPSV and acknowledges complex relationships between IPSV and psychological abuse. In other words, services must respond to the uniquely harmful and sexualised nature of IPSV and avoid treating all forms of IPV the same. In addition to the need to be listened to, empathised with, and validated, IPSV survivors need service providers to address the unique context of betrayal, disorientation, shame and silencing associated with IPSV. Yet, despite needing more targeted support, survivors’ responses indicate their disclosures of IPSV were even *more* likely to be dismissed, overlooked and disregarded. It is critical that service providers address the specific dynamics of IPSV to avoid silencing IPSV survivors further when they seek support.

## Data Availability

The datasets used and/or analysed during the current study are available from the authors on reasonable request.

## Abbreviations

IPSV: Intimate Partner Sexual Violence
IPV: Intimate Partner Violence
SV: Sexual Violence
